# Draft Genome Sequence of Enterococcus faecalis Strain OB15, a Probiotic Strain Recently Isolated from Tunisian Rigouta Cheese

**DOI:** 10.1128/MRA.01433-19

**Published:** 2020-04-09

**Authors:** Olfa Baccouri, Amine M. Boukerb, Flore Nilly, Mélyssa Cambronel, Issam Smaali, Marc G. J. Feuilloley, Ferid Abidi, Nathalie Connil

**Affiliations:** a Laboratory of Protein Engineering and Bioactive Molecules, National Institute of Applied Sciences and Technology, University of Carthage, Tunis, Tunisia; b Laboratoire de Microbiologie Signaux et Microenvironnement, EA 4312, Université de Rouen Normandie, Normandie Université, Évreux, France; Broad Institute

## Abstract

Enterococcus faecalis OB15 is a probiotic strain that was isolated from rigouta, a popular traditional Tunisian fermented cheese. We report here the draft genome sequence of this strain, consisting of 2,912,159 bp, with an average G+C content of 37.49%.

## ANNOUNCEMENT

Enterococcus faecalis is a Gram-positive, diplococcus-forming, catalase-positive, and facultative anaerobic bacterium with typical cream-colored colonies on MRS agar. This microorganism is ubiquitous in the environment; it is found mainly in the gastrointestinal tracts of humans and animals but is also widespread in soil, water, plants, and dairy products (1). Several strains are known to be opportunistic pathogens and are recognized among the major etiological agents of nosocomial and other human infections (endocarditis and urinary tract infections), due to their ability to acquire resistance to a wide range of antibiotics and the presence of virulence determinants (2, 3). In contrast, other strains have potential benefits for human health and are currently used as starter cultures in manufacturing cheese (4, 5), as bacteriocin (enterocins) producers in food preservation, and as potential probiotics in the food industry (1, 6).

Recently, we isolated E. faecalis OB15 from rigouta, a popular traditional Tunisian fermented cheese that is often prepared by boiling acidified cheese whey from cow’s milk (7). Physiological and genomic analyses demonstrated that E. faecalis OB15 has met all of the principle requirements for and has properties of an efficient probiotic and could be a reliable candidate for future use in the food or feed industry (7).

Genomic DNA of E. faecalis OB15 was extracted from an overnight culture in MRS broth at 37°C, under static conditions, with the GeneJET genomic DNA purification kit (Thermo Fisher Scientific, France), following the manufacturer’s recommendations. Extracted DNA was quantified using a Qubit fluorometer and the double-stranded DNA (dsDNA) high-sensitivity (HS) assay kit (Thermo Fisher Scientific). Libraries were prepared using the Nextera XT DNA sample kit (Illumina, San Diego, CA), and genomic sequencing was performed using a MiSeq instrument (Illumina) with a 2 × 250-bp paired-end protocol (Laboratoire de Microbiologie Signaux et Microenvironnement, Université de Rouen Normandie, Normandie Université, Évreux, France).

Default parameters were used except where otherwise indicated. The 3,302,996 reads obtained were trimmed using Trim Galore v.0.6.2 (8), and their quality was checked with FastQC v.1.6 (9). *De novo* assembly was performed with Unicycler v.0.4.7 (10), and QUAST v.5.0.0 (11) was used to check assembly consistency (e.g., the number of contigs, G+C content, *N*_50_ value, and total size.). Genome annotation was carried out using the Prokka pipeline v.1.14.0 (12). The multilocus sequence typing (MLST) profile of OB15 was determined from the draft genome sequence using the software package MLST v.2.16.1 (13), based on the E. faecalis PubMLST database (https://pubmlst.org/efaecalis). Antimicrobial mechanisms (e.g., enterocin production) were explored with BAGEL v.4 (14) and antiSMASH v.5 (15).

The draft genome assembly has a mean coverage of 204× and consists of 2,912,159 bp, with a mean G+C content of 37.49%, which is consistent with that of published E. faecalis genomes (https://www.ncbi.nlm.nih.gov/genome/genomes/808). The Unicycler assembler yielded 80 contigs, with an *N*_50_ value of 93,106 bp; the largest contig was 256,721 bp. A total of 2,820 coding sequences, 52 tRNA genes, and 3 rRNA genes were predicted. MLST analysis identified OB15 as sequence type 25. BAGEL4 and antiSMASH5 did not allow us to predict any previously described bacteriocin biosynthesis gene.

### Data availability.

The draft genome sequence of E. faecalis OB15 has been deposited at DDBJ/ENA/GenBank under accession number VOLU00000000. The version described in this paper is the first version, VOLU00000000.1. Raw sequence reads have been deposited under accession number SRR9859820.
